# MiRNA-34a, miRNA-145, and miRNA-222 Expression, Matrix Metalloproteinases, TNF-α and VEGF in Patients with Different Phenotypes of Coronary Artery Disease

**DOI:** 10.3390/ijms252312978

**Published:** 2024-12-03

**Authors:** Alfiya Oskarovna Iusupova, Nikolay Nikolaevich Pakhtusov, Olga Alexandrovna Slepova, Natalia Vladimirovna Khabarova, Elena Vitalievna Privalova, Irina Vladimirovna Bure, Marina Vyacheslavovna Nemtsova, Yuri Nikitich Belenkov

**Affiliations:** 1 Department of Hospital Therapy No 1, I.M. Sechenov First Moscow State Medical University (Sechenov University), 119048 Moscow, Russia; pakhtusovnn@gmail.com (N.N.P.); slepova_o_a@staff.sechenov.ru (O.A.S.); khabarova_n_v@staff.sechenov.ru (N.V.K.); privalova_e_v@staff.sechenov.ru (E.V.P.); belenkov_yu_n@staff.sechenov.ru (Y.N.B.); 2 Laboratory of Medical Genetics, I.M. Sechenov First Moscow State Medical University (Sechenov University), 119048 Moscow, Russia; bureira@mail.ru (I.V.B.); nemtsova_m_v@mail.ru (M.V.N.); 3 Research Institute of Molecular and Personalized Medicine, Russian Medical Academy of Continuous Professional Education, I.M. Sechenov First Moscow State Medical University (Sechenov University), 119048 Moscow, Russia; 4 Laboratory of Epigenetics, Research Centre for Medical Genetics, 115522 Moscow, Russia

**Keywords:** microRNA (miR-34a: miR-145: miR-222), matrix metalloproteinases (MMP-1: MMP-9: MMP-13: MMP-14), tumor necrosis factor α (TNF-α), vascular endothelial growth factor (VEGF), ischemia/angina with non-obstructive coronary arteries (INOCA/ANOCA), coronary artery disease (CAD)

## Abstract

The development of different phenotypes of coronary artery (CA) lesions is regulated via many various factors, such as pro-inflammatory agents, zinc-dependent endopeptidases, growth factors and circulating microRNAs (miRs). To evaluate the expression levels of miR-34a, miR-145 and miR-222, tumor necrosis factor α (TNF-α), matrix metalloproteinases (MMP-1, -9, -13 and -14) and vascular endothelial growth factor (VEGF) in patients with different phenotypes of coronary artery disease (CAD): ischemia/angina with non-obstructive coronary arteries (INOCA/ANOCA) and obstructive CAD (oCAD) compared with a control group. This cross-sectional observational study included 157 subjects with a verified CAD diagnosis (51 patients with INOCA, 76 patients with oCAD and 30 healthy volunteers). The expression of miR-34a, miR-145 and miR-222 (RT-PCR) and the levels of VEGF, TNF-α, MMP-1, MMP-9, MMP-13 and MMP-14 (ELISA) were estimated in plasma samples. A higher concentration of MMP-9 was found in oCAD-group samples compared to the INOCA/ANOCA group. The INOCA/ANOCA group was characterized by higher levels of TNF-α. Based on multivariate regression analysis, a mathematical model predicting the type of CA lesion was constructed. MiR-145 was the independent predictor of INOCA/ANOCA (*p* = 0.006). Changes in concentrations of MMP-9 and MMP-14 were found in both investigated CAD groups, with MMP-9 levels being significantly higher in obstructive CAD samples than in INOCA/ANOCA, which confirms the role of inflammation in the development of atherosclerosis. A multivariate regression analysis allowed us to achieve a model that can predict the phenotype of stable CAD, and MiR-145 can be assumed as an independent predictor of INOCA/ANOCA.

## 1. Introduction

The prevalence of metabolic risk factors and the aging of the population in developed countries lead to the persistence of high mortality rates from non-communicable diseases and from coronary artery disease (CAD) in the world [1]. According to the World Health Organization (WHO) in 2021, the mortality rate from CAD reached 17.9 million people [2]. Among patients with CAD, there are phenotypes with ischemia/angina and non-obstructive coronary arteries (INOCA/ANOCA), the prevalence of which, in the population with CAD, according to some studies, reaches 13% [3]. At the same time, INOCA/ANOCA is significantly more common in women (61%, and, in accordance with other data—65%) than in men (32%) [4,5,6,7]. The development of different types of CA lesions is regulated via many various factors, among which pro-inflammatory agents play a significant role. According to a study by Karakayali M. et al., the systemic immuno-inflammatory index (SII, platelet × neutrophil/lymphocyte ratio) is independently associated with the presence of INOCA [8]. It is known that, in the case of INOCA, an increase in the systemic inflammation response index (SIRI) plays a potentially important role in the diagnosis of the slow coronary flow phenomenon [9]. Additionally, numerous factors—including pro-inflammatory cytokines (IL and TNF-α), reactive oxygen species (ROS), an imbalance between vasodilation (NO) and vasoconstriction mediators, several zinc-dependent endopeptidases (matrix metalloproteinases, MMP) and growth factors (such as VEGF)—influence the progression of CA atherosclerosis. This effect was particularly demonstrated in the study by Beatty et al., which showed that TNF-α induces the activation of macrophages, T-helper 1 lymphocytes and endothelial cells in atherosclerosis. Activated endothelial cells produce receptors and adhesion molecules that promote abnormal leukocyte migration into the vascular wall, leading to the dysregulated secretion of pro-inflammatory cytokines. The authors conclude that TNF-α and IL-6 play significant roles as risk factors for the development of CAD [10].

MMPs are zinc-dependent endopeptidases, which consist of pro-peptide, the C-terminal domain and the catalytic domain [11]. MMPs are synthesized in the latent form and regulated via a number of proinflammatory factors. MMPs are involved in the processes of extracellular matrix degradation [12].

The classification of MMPs is based on substrate specificity and sequence similarity. Among the groups of MMPs, there are collagenases, gelatinases, stromelysins, matrilysins, membrane-type MMPs and others [13,14]. It is known that foam and smooth muscle cells (SMCs) produce MMPs, which play a significant value in the formation and growth of atherosclerotic plaques [15]. At the same time, MMPs produced by macrophages are implicated in the process of fibrous-cap thinning and plaque destabilization [16]. Thus, the increased expression of activated MMP-13 is related to increased collagenolysis in vivo, mainly in atheromatous plaques, and a decrease in the accumulation of SMC at different stages of atherogenesis, in particular, at the preclinical stage [17]. The investigation by Lehrke M. et al. demonstrated that the level of circulating MMP-1 can be used as a predictive marker of the presence of atherosclerotic plaques [18].

The study of the significance of circulating microRNAs (miRs) in the epigenetic regulation of the development and progression of atherosclerosis is also of considerable interest. MiRs are short non-coding RNA molecules that modify gene expression through the inhibition of translation or enhancing mRNA destruction. Recent research highlights the significance of miRNAs as promising diagnostic biomarkers for assessing the severity of CA stenosis. In particular, the expression of miR-34a increases in the heart during myocardial hypertrophy caused by stretching, adrenergic stimulation and hypoxia, which has been shown to contribute to the disruption of angiogenesis by uncoupling hypoxia-inducible factor 1-α–VEGF (HIF-1α-VEGF) signaling [19]. In addition, it is known that miR-34a, miR-92a, miR-181c and miR-210 participate in the regulation of endothelial function of the vascular wall by inducing or reducing the level of oxidative stress markers [20,21].

The expression of miR-145 decreases in acute coronary syndrome (ACS), which makes it possible to consider this miR as a potential diagnostic biomarker. A negative correlation of miR-145 with markers of endothelial damage (von Willebrand factor (vWF) and heart-type fatty acid-binding protein (H-FABP)) and pro-inflammatory cytokines (IL-6 and TNF-α) was also found [22]. In turn, miR-221 and miR-222 participate in the processes of control of endothelial inflammation, angiogenesis and the apoptosis of endothelial cells [23].

According to the literature data, there is isolated evidence on the expression of miR-34a, miR-145 and miR-222 in patients with CAD, including those with INOCA/ANOCA. It should be noted that, in patients with CAD (obstructive and non-obstructive phenotypes), no investigation was conducted on the possible association of the expression of these miRs with pro-inflammatory cytokines (TNF-α), MMPs and growth factors (VEGF). The study of miR expression levels in such patients can clarify some aspects of the atherogenesis in CA and, in consequence, may be important for determining their potential as possible diagnostic biomarkers and for the development of new therapies with antagomiRs.

## 2. Results

### 2.1. Basic Clinical Characteristics

**Figure 1:**
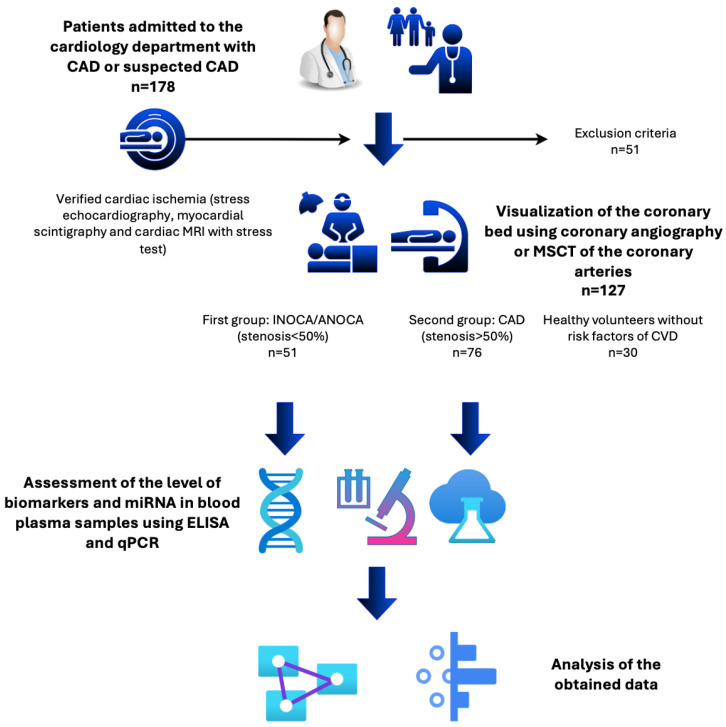
The design of the study. CAD—coronary artery disease; INOCA/ANOCA—ischemia/angina with non-obstructive coronary arteries; qPCR—quantitative polymerase chain reaction; MRI—magnetic resonance imaging; MSCT—multi-detector computed tomography.

Plasma samples were collected from patients admitted to the cardiology department presenting with complaints of chest pain and dyspnea. All participants underwent stress tests, including stress echocardiography, myocardial scintigraphy, and cardiac MRI with stress, to elucidate the diagnosis of coronary artery disease (CAD). In patients with identified myocardial ischemia, coronary artery imaging (coronary angiography or MSCT) was additionally conducted to assess the necessity for revascularization. The study comprised 127 patients categorized into two groups based on the extent of coronary artery obstruction: the first group included 51 patients with hemodynamically insignificant stenosis (INOCA/ANOCA, stenosis less than 50%), while the second group included 76 patients with coronary artery obstruction (obstructive CAD, stenosis exceeding 50%). The control group consisted of 30 healthy volunteers devoid of risk factors for cardiovascular diseases. The design of the study is presented in Figure 1.

The general clinical and demographic characteristics of the groups are summarized in Table 1 [24]. The groups included in the study were comparable according to the main clinical and demographic indicators (age and body mass index (BMI)). In the INOCA/ANOCA group, the majority were women (60.8% women vs. 39.2% men).

In the group with hemodynamically significant CA stenosis, the levels of total cholesterol and LDL were lower than in the INOCA/ANOCA group. We assume that these results can be explained by better control of lipid spectrum parameters due to the administration of higher doses of statins.

The therapy that patients received at the time of hospitalization is summarized in Table 2.

### 2.2. Concentration of MMPs, TNF-α and VEGF in Plasma

Statistically significant differences were revealed when the levels of MMPs-9 and -14 and TNF-α were compared in the study groups and the control group. The level of VEGF was significantly higher only in the INOCA/ANOCA group compared to the control group. Also, we found differences between the different phenotypes of CAD in the case of TNF-α and MMP-9.

The highest level of MMP-9 was observed in the group with significant stenoses of CA. In addition, in this group, concentrations of VEGF and TNF-α were lower when compared with INOCA/ANOCA. The differences in MMP-1 and MMP-13 levels in both groups were statistically insignificant (Table 3) [24].

### 2.3. MiRNA Expression in Plasma

**Figure 2:**
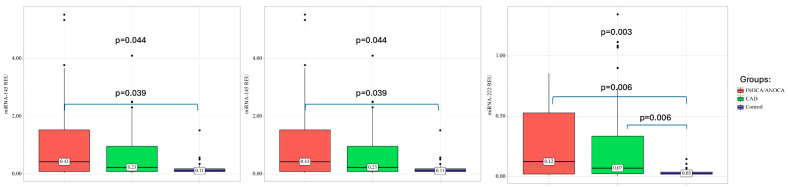
MiRNAs expression in plasma of CAD patients and healthy volunteers (control). All values are presented as the median and CI. Statistically significant—*p* < 0.05; CAD—coronary artery disease, INOCA/ANOCA—ischemia/angina with non-obstructive coronary arteries.

In patients with different phenotypes, CAD expression of miR-34a (*p* < 0.001) and miR-222 (*p* < 0.001) was higher than in the control group. Analyzing the expression of miR-145 can establish that significant differences from the control are present only in the INOCA group (*p* = 0.039) (Figure 2) [24].

### 2.4. Correlations of VEGF, TNF-α and MMPs with Circulating miRNAs

According to the correlation analysis, in the INOCA/ANOCA group, notable associations were recorded between VEGF and MMP-1 (*p* = 0.668; *p*< 0.001), as well as MMP-14 (*p* = 0.629; *p*< 0.001). In the obstructive CAD group, a moderate association was observed between VEGF and MMP-9 (ρ = 0.400; *p*< 0.001) and a strong association between MMP-1 (ρ = 0.825; *p*< 0.001) and MMP-14 (ρ = 0.736; *p*< 0.001).

In the INOCA/ANOCA group, there were observed moderate correlations of miR-145 with VEGF (ρ = 0.442; *p* = 0.013) and TNF-α (negative relationship, ρ = −0.386; *p* = 0.032) and a strong negative linkage with MMP-13 (ρ = −0.729; *p*< 0.001). A moderate positive association of miR-222 with VEGF (ρ = 0.414; *p* = 0.021), a significant correlation with MMP-1 (ρ = 0.652; *p* < 0.001) and a strong correlation with MMP-14 (ρ = 0.701; *p* < 0.001) were also found.

In the obstructive CAD group, a significant correlation of miR-145 with VEGF (ρ = 0.584; *p* < 0.001) and a strong negative association with MMP-13 (ρ = −0.380; *p* = 0.002) were observed. In addition, a moderate association of miRNA-222 with TNF-α (ρ = 0.363; *p* = 0.004) and a significant correlation with VEGF (ρ = 0.595; *p* < 0.001), as well as a strong linkage with MMP-1 (ρ = 0.726; *p* < 0.001) and with MMP-14 (ρ = 0.727; *p* < 0.001) were demonstrated.

Univariate logistic regression determined expression of miR-145 as significant predictor of INOCA/ANOCA (Table 4).

The mathematical model was created based on multivariate regression analysis. Based on the obtained data, it can be assumed that miRNA-145 may be an independent predictor of INOCA/ANOCA (Table 5).

**Figure 3:**
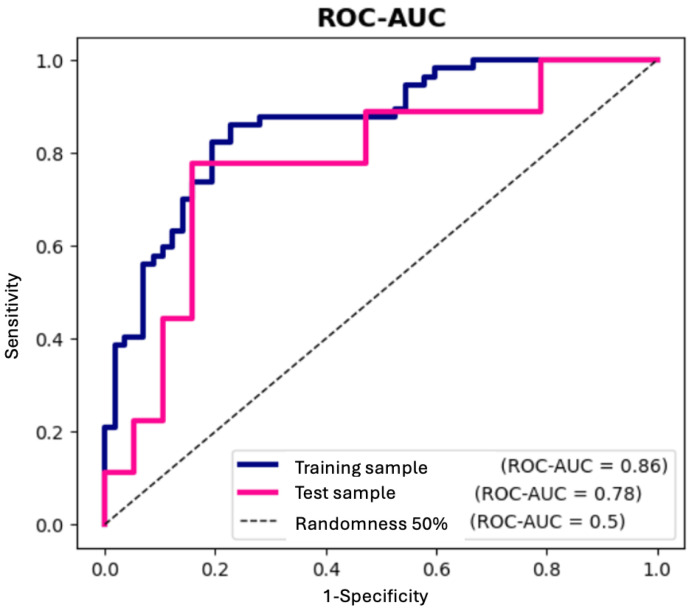
Diagnostic value analysis of the model by ROC curve.

The probability of INOCA/ANOCA can be proposed according to the results of an ROC analysis ((cut-off = 0.48) with sensitivity of 77.8% [50.0; 100.0]%, specificity of 78.9% [62.5; 93.3]% and ROC-AUC = 77.8% [60.2; 93.8]%) (Figure 3).

## 3. Discussion

The development and progression of CA atherosclerosis in stable CAD is influenced by many independent factors that play a crucial role in the formation of different variants of CA lesions. One of these factors is angiogenesis, in the regulation of which VEGF is actively involved. Several members of VEGF family participate in the processes of atherogenesis. In previous studies, it has been demonstrated that VEGF can influence the growth and instability of atherosclerotic plaque through angiogenesis-independent processes [25,26].

It is known that, in metabolic syndrome, the activity of angiogenesis processes in CA may be reduced, despite the increased expression of VEGF, which is associated with an altered VEGF signaling pathway—VEGF/VEGFR signaling [27]. VEGF is not only involved in angiogenesis, stimulating the proliferation and growth of endothelial cells, but also affects pro-inflammatory cytokines and cells: it increases the levels of TNF-α and IL-1 in monocytes and reduces IL-6. Similar results were obtained in our study from plasma samples with obstructive CAD [28].

TNF-α is predominantly produced by activated macrophages and many other types of cells. Recent studies have demonstrated that TNF-α and VEGFA gene expression was significantly lower in patients with INOCA compared to patients with obstructive CAD [29].

In addition, the expression of MMP-1, MMP-3 and MMP-13 can be enhanced through VEGF [30]. The risk of CAD and various severities of the disease may have a relationship with changes in MMP levels. Our study revealed significant differences between levels of MMP-9 and MMP-14 in samples with different phenotypes of CAD compared to the control group. Direct correlations of VEGF with MMP-1, MMP-9 and MMP-14 were found in obstructive CA lesions, and only MMP-1 and MMP-14 with non-obstructive ones.

According to Buchler A., et al. and Quillard T., et al., the increased expression of activated MMP-13 is associated with increased collagenolysis in vivo, which accelerates vascular remodeling processes in atherosclerotic lesions [17,31]. In the study of Gaubatz J. W. et al. a positive relationship between plasma levels of MMP-1 and the calcification of the carotid arteries was revealed [32]. At the same time, in the study of Polonskaya Y.V. et al. the levels of MMP-1 and MMP-9 were increased in calcified plaques, but according to logistic regression analysis, the relative risk of CA plaque calcification was associated only with MMP-9 [33].

MMP-9 is also associated with plaque instability, while MMP-13 is involved in the initial and later stages of atherogenesis [34]. MMP-13 promotes the degradation of the extracellular matrix, thus leading to plaque destabilization [31,35].

Currently, the role of MMP-14 in the development of CAD has been proven in many studies. Thus, high levels of MMP-14 were independently correlated with CVD [36]. An increased expression of MMP-14 was detected by Johnson J.L. et al. in the foam cells of unstable atherosclerotic plaques [37].

In our study, the absence of significant differences between the groups in MMP-1 and MMP-13 levels may be due to the fact that MMP activity was determined only in stable CAD, and the inclusion of a group with ACS may help reveal these differences.

It has been established that miRs are involved in the regulation of various processes, among which the most important in atherogenesis are endothelial damage, inflammation development, the activation of macrophages, the formation of foam cells in the vascular wall, the proliferation, migration and apoptosis of SMCs and, as a consequence, vascular remodeling [16,38]. Epigenetic regulation, in particular microRNAs, changes with age, and some microRNAs undergo an age-dependent decrease, while the expression of others, on the contrary, increases during life. The younger age and demographic characteristics of the control group are due to the fact that we recruited healthy volunteers without risk factors for cardiovascular diseases (for example, those without obesity and overweight, those without acute and chronic diseases and non-smokers).

In the present study, the level of expression of miR-145 in the groups significantly differed and reached the highest values in patients with INOCA/ANOCA. It should be noted that a negative high-strength correlation was found between miR-145 and MMP-13. According to available scientific data, miR-145-5p can act as a “cardioprotective” molecule in patients with myocardial ischemia and, through the negative regulation of CD40, is involved in reducing the activity of the inflammatory response and apoptosis caused by hypoxia [39]. The overexpression of miR-145 can eliminate endothelial damage and reduce the activity of abnormal inflammation, which makes it possible to consider this miR as a potential candidate for targeted ACS therapy [22].

Different phenotypes of CAD can be united by the presence of endothelial dysfunction. It can be hypothesized that INOCA/ANOCA represents an initial stage in the progression of classical CAD. The anti-atherogenic miRNA-145 may function as part of a compensatory mechanism protecting vascular walls against stenotic atherosclerosis.

In the study by Wu S. et al., a negative relationship between miR-145 expression and levels of vWF, H-FABP, IL-6 and TNF-α was demonstrated. Thus, the authors concluded that the reduced expression of miR-145 in serum can serve as a potential diagnostic biomarker of ACS [22]. In our study, similar results were obtained in the INOCA/ANOCA group: a moderate negative correlation between miR-145 and TNF-α was revealed. According to the work of Zhang X. et al., miR-145-5p expression was reduced in blood samples and arterial walls of patients with stenotic atherosclerosis of the CA [40]. Kumar A. et al. found that lower levels of miR-145 and miR-155, along with endothelial dysfunction, were associated with the severity of CKD and with a higher risk of CVD development [41]. Thus, our data support the assumption that a lower level of miR-145 expression in obstructive CAD, in contrast to INOCA, may be associated with a more severe atherosclerotic lesion of CA.

As for the analysis of miR-34a, the minimal level of expression of this miR was observed in the control group, represented by healthy volunteers without CVD risk factors, while in obstructive and non-obstructive CA lesions, the expression was significantly higher. Similar results were obtained by Gatsiou A. et al.: high miR-34a levels were independently associated with the presence of CAD, while the simultaneous overexpression of miR-34-a/c or all three miR-34a/b/c was associated with increased stiffness of the aorta [42].

MiR-34a promotes the downregulation of SIRT1 and VEGF protein expression, causing a disruption of angiogenesis processes and inducing cell death [19]. A study of the role of miR-21, -34a, -146a, -146b-5p and -210 demonstrated a significant increase in their expression in atherosclerotic plaques in contrast to intact arteries [43]. All of these data confirm a significant upregulation in miR-34a expression levels in the plasma of patients with CAD compared to the control group [44].

Similar results were obtained from studying miR-222, the expression of which was significantly higher in both study groups when compared with the control. According to available scientific data, miR-222 also takes part in atherogenesis. MiR-221/222 and miR-155 suppress the inflammatory response induced via angiotensin II in endothelial cells, affecting transcription factor Ets-1 and the genes vascular cell adhesion molecule 1 (VCAM-1) and monocyte chemoattractant protein-1 (MCP1) [45]. The overexpression of miR-19b-3p, miR-221-3p and miR-222-3p induced via inflammatory cytokines TNF-α and IFNγ resulted in intracellular ROS accumulation, leading to cell apoptosis [46]. In obstructive CAD, a moderate association between miR-222 and TNF-α expression was found, as well as a high-strength correlation with MMP-1 and MMP-14 [47]. Karere G.M. et al. identified vascular miRs expressed in baboons and humans and potential novel miRs associated with atherosclerosis. The expression of miR-144-3p, miR-146a-5p, miR-21 and miR-221/222-3p was increased in fibrous plaques, while miR-195-5p was decreased. These results indicate that some miRs are not only specific to a particular type of lesion but also exhibit different levels of expression at various stages of the disease. This indicates their potential importance in the initiation and development of atherosclerosis [48].

In our study, according to the results of multivariate regression analysis, miR-145 was identified as an independent predictor of the non-obstructive phenotype of stable CAD.

In accordance with the results of the ROC analysis, it was concluded that various miRs can be considered potential diagnostic noninvasive markers of CAD. Multivariate logistic regression analysis revealed that levels of circulating miR-145 and miR-155 were associated with CAD and may be powerful markers for detecting CAD [49]. O’Sullivan J. et. al. concluded that four miRNAs (miR-15a-5p, miR-146a-5p, miR-16-5p and miR-93-5p) were predictors of stable CAD development [50]. Zhang L. et. al. suggested other miRs (miR-29a-3p, miR-574-3p and miR-574-5p with AUCs 0.83, 0.792 and 0.789, respectively) as potential markers for the noninvasive diagnosis of CAD [51].

Recent data suggest that plasma levels of miR-23b and miR-143 may be useful as non-invasive biomarkers for in-stent restenosis [52]. Gholipour A. et al. established the potential value of miR-6721-5p as a biomarker for CAD [53].

Our results can be useful in the comprehension of potential roles of miRs, and we plan to continue our research on the mechanisms of the epigenetic regulation of the pathogenesis of different phenotypes of CAD. An investigation of the pathogenesis of atherosclerotic CA lesions may be important for determining the potential role of miRs as possible diagnostic biomarkers and in the development of new treatment methods using antagomiRs. It is necessary to continue careful study and fundamental research in order to solve this complicated problem.

## 4. Materials and Methods

### 4.1. Patient Population

This article represents a continuation of our efforts within a grant project [24]. Over the course of the year, we recruited an additional cohort of patients and augmented the number of samples in all groups, except for the control group. As of the current writing, 127 individuals who met the inclusion criteria and provided informed consent from 2020 to 2024 have been enrolled in the ongoing cross-sectional observational study. The study was conducted in accordance with the principles of the Declaration of Helsinki, and it received approval from the Ethics Committee of Sechenov University (Protocol No. 01-21, dated 22 January 2021).

The participants in the study consisted of men and women aged 45 to 75 years who had been diagnosed with CAD. The diagnosis of myocardial ischemia in hospitalized patients with stable angina or its equivalents was performed using instrumental methods, including stress echocardiography (EchoCG) and single-photon emission computed tomography (SPECT), conducted in conjunction with stress tests. Based on the outcomes of coronary angiography (CAG) or multispiral computed tomography (CT), the patients were categorized into two groups: the first group (non-obstructive coronary artery disease, characterized by stenosis < 50% or unchanged arteries) increased from 20 to 51 individuals, while the second group (obstructive coronary artery disease with hemodynamically significant stenosis) expanded from 44 to 76 individuals. The control group, consisting of 30 healthy volunteers without cardiovascular diseases and risk factors, remained unchanged.

The exclusion criteria were as follows: diabetes mellitus, acute coronary syndrome, myocardial infarction or stroke within the preceding three months, chronic heart failure of functional class III-IV (as per the NYHA classification), autoimmune and oncological diseases, decompensated liver diseases, portal hypertension, uncontrolled bronchial asthma, chronic obstructive pulmonary disease, acute gastric or duodenal ulcers, acute chronic pancreatitis, malignant neoplasms, thyroid pathologies, Cushing’s syndrome, acute or terminal renal failure (GFR < 15 mL/min/1.73 m^2^), mental disorders, alcoholism, drug addiction, substance abuse, pregnancy and breastfeeding.

### 4.2. Collection of Blood Samples and ELISA

Blood plasma samples were collected in tubes with EDTA K3 as an anticoagulant, centrifuged for 20 min at 1000× *g* (1000 RCF) and further frozen in cryotubes at −80 °C. To estimate the MMPs’ levels, a VEGF and TNF-α enzyme immunoassay (ELISA) was performed using the ELISA analyzer of Adaltis Personal Lab (Rome, Italy) and Wuhan Fine Biotech Co., Ltd. kits (Wuhan, China) (catalog numbers: EH0232, EH0238, EH0234, EH0369, EH0302 and EH0327). The coefficient of variation (CV) for the sets was 10% and 12%, respectively. All patients had undergone standard biochemical tests, including indicators of the lipid spectrum, glucose and uric acid.

### 4.3. RNA Extraction and Reverse-Transcription–Polymerase Chain Reaction (RT-PCR) Assay

Total RNA was extracted from blood samples using Qiazol reagent (Qiagen, Hilden, Germany), following the protocol of the manufacturer with small modifications. The concentration and purity of the obtained RNA was estimated on the NanoDrop 2000 microvolume spectrophotometer (Thermo Fisher Scientific, New York, NY, USA). The process of extraction was repeated for each sample until a sufficient amount of RNA was obtained.

cDNA was synthesized from 300 ng of total RNA using MiScript II RT Kit (Qiagen) under the recommended protocol. Real-time PCR was performed on the CFX96 Real-Time PCR Detection System (Bio-Rad, Hercules, CA, USA) in three repetitions for each transcript and control, using the MiScript SYBR Green PCR Kit (Qiagen) according to the manufacturer’s recommended protocol. Primers for all three investigated miRNAs were designed according to the instructions (Table 6) [54]. The presynthesized MiScript Primer Assay (Ce_miR-39_1, identification code MS00019789, Qiagen, Germany) was used for the exogenous control, cel-miR-39-3p. Data of the ncRNAs’ expression were normalized and analyzed using the 2^−∆∆Ct^ method. The results are presented as REU (relative units of expression).

### 4.4. Statistical Analysis

The data presented in this paper are provided in the figures and tables. The statistical analysis of the results was performed using the program StatTech v.v. 3.1.10 (StatTech, Kazan, Russia) and the free Python computing software environment (v.3.11).

#### 4.4.1. Clinical Data Analyses

Demographic, clinical and laboratory data were compared via either a Kruskal–Wallis test, Dunn test and Fisher’s exact test for categorical variables. The two groups were compared quantitatively with an abnormal distribution using the Mann–Whitney U-test.

#### 4.4.2. Multiple Logistic Regression

Multiple logistic regression (MLR) was used to build a model for predicting the presence/absence of a characteristic. The choice of the method was based on the dichotomy of the dependent variable and the fact that independent variables characterize both categorical and quantitative characteristics. The independent variables were selected through step-by-step reverse selection using Wald statistics as an exclusion criterion. The statistical significance of the obtained model was determined using the criterion χ^2^. To balance the training data set for a more stable predictive model, the SMOTE (Synthetic Minority Over-sampling Technique) method was employed. To estimate the quality of the constructed model, the following criteria were used: ROC-AUC, accuracy, sensitivity, specificity, and DCA analysis (decision curve analysis). Metrics were calculated together with the 95% CI. The 95% CI was calculated using the bootstrap method with a sample of 1000 instances. The threshold value was chosen in accordance with the maximization of sensitivity and specificity.

### 4.5. Study Limitations

This study involved potential limitations. According to the European clinical guidelines for chronic coronary syndromes (2024), the incidence of INOCA/ANOCA is higher in women. So, this fact limited our sample size and the ability to obtain fully comparable study groups. The control group included healthy volunteers without any risk factors for cardiovascular diseases (for example, those without acute and chronic diseases, obesity, overweight and non-smokers) who were younger than the main groups of patients with different phenotypes of CAD.

## 5. Conclusions

The aim of this study was to evaluate the expression levels of miR-34a, miR-145 and miR-222, TNF-α, MMP-1, -9, -13 and -14, and VEGF in order to search for possible diagnostic markers of different phenotypes of CAD. Changes in MMP levels may be associated with an increased risk of the development of CAD and varying severity of the disease. In our study, MMP-9 levels were significantly different between the groups with obstructive CAD and INOCA/ANOCA. Direct correlations of VEGF with MMP-1, MMP-9 and MMP-14 were found in obstructive CAD, versus only MMP-1 and MMP-14 in INOCA/ANOCA.

MiRs can be considered potential diagnostic markers of stable CAD. The multivariate regression analysis allowed us to achieve a model that can predict the phenotype of coronary artery lesions with high sensitivity and specificity among patients with stable CAD. Thus, we can assume that miR-145 can be potentially used as a predictor of INOCA/ANOCA.

## Figures and Tables

**Table 1:** Basic clinical characteristics.

	All CAD (n = 127)	INOCA/ANOCA (n = 51)	Obstructive CAD (n = 76)	Control (n = 30)	*p*-Value
Men (%)	71 (57.4)	20 (39.2)	51 (67.1)	10 (33.3)	0.001 * p_INOCA/ANOCA–obstructive CAD_ = 0.004 *p*_obstructive CAD–Control_ = 0.003
Women (%)	56 (42.6)	31 (60.8)	25 (32.9)	20 (66.7)	
Age (year)	64 [57; 71]	64 [59; 70.5]	63 [56; 71]	28.5 [26; 39.2]	<0.001 * *p*_control–INOCA/ANOCA_ < 0.001 *p*_control–obstructive CAD_ < 0.001
BMI (kg/m^2^)	27.4 [25; 30.2]	26.20 [25.67; 30.40]	27.4 [24.77; 29.75]	21.95 [20.75; 25.23]	<0.001 * *p*_control–INOCA/ANOCA_ < 0.001 *p*_control–obstructive CAD_ < 0.001
Smoking (%)	9 (7.8)	3 (7.7)	6 (7.9)	-	0.953
Hemoglobin (g/L)	144 [135; 155]	142 [134; 151]	144 [133; 152]	136 [129; 152]	0.459
Glucose (mmol/L)	5.45 ± 0.54	5.53 [5.25; 5.81]	5.43 [5.31; 5.54]	4.9 [4.67; 5.35]	0.005 * p_INOCA/ANOCA–Control_ = 0.011 *p*_obstructive CAD–Control_ = 0.007
Creatinine (µmol/L)	89 [77.5; 99.15]	80.45 [72.08; 91.67]	91.5 [81; 101.32]	82 [77.7; 87]	0.009 * p_INOCA/ANOCA–obstructive CAD_ = 0.023
Total cholesterol (mmol/L)	4.05 [3.45; 5.21]	4.45 [3.49; 5.36]	3.79 [3.25; 4.36]	4.94 [4.39; 5.52]	<0.001 * *p*_INOCA/ANOCA–obstructive CAD_ = 0.015 *p*_obstructive CAD–Control_ < 0.001
LDL (mmol/L)	2.35 [1.81; 2.99]	2.72 [2.03; 3.2]	2.16 [1.58; 2.55]	2.54 [2.28; 3.21]	0.006 * *p*_obstructive CAD–INOCA/ANOCA_ = 0.016 *p*_control – obstructive CAD_ = 0.044
HDL (mmol/L)	1.24 ± 0.47	1.31 [1.03; 1.46]	1.08 [1.08; 1.32]	1.62 [1.35; 1.9]	<0.001 * *p*_control–INOCA/ANOCA_ = 0.021 *p*_control–obstructive CAD_ < 0.001

* Statistically significant—*p* < 0.05; n—number of patients in the group; CAD—coronary artery disease; INOCA/ANOCA—ischemia/angina with non-obstructive coronary arteries; BMI—body mass index; LDL—low-density lipoproteins; HDL—high-density lipoproteins.

**Table 3:** The concentration of VEGF, TNF-α and MMPs in plasma.

Proteins	Groups	Concentration (Me [Q1–Q3])	*p*-Value
VEGF, ng/mL	INOCA/ANOCA	41.66 [36.23–47.58]	0.043 * p_INOCA/ANOCA–control_ = 0.036
	Obstructive CAD	36.4 [13.12–66.05]	
	Control	35.03 [10.50–41.62]	
TNF-α, ng/mL	INOCA/ANOCA	28.33 [13.97–29.74]	<0.004 * p_control–obstructive CAD_ = 0.037 *p*_INOCA/ANOCA– obstructive CAD_ = 0.03
	Obstructive CAD	13.85 [10.76–25.30]	
	Control	28.23 [14.17–28.73]	
MMP-1, ng/mL	INOCA/ANOCA	0.21 [0.17–0.29]	0.161
	Obstructive CAD	0.23 [0.21–0.23]	
	Control	0.24 [0.22–0.32]	
MMP-9 ng/mL	INOCA/ANOCA	3.58 [1.98–6.18]	<0.001 * p_obstructive CAD–INOCA/ANOCA_ < 0.001
	Obstructive CAD	7.2 [4.25–10.68]	
	Control	5.45 [4.02–6.81]	
MMP-13, ng/mL	INOCA/ANOCA	123.95 [68.85–285.43]	0.055
	Obstructive CAD	91.57 [49.77–339.51]	
	Control	67.5 [47.79–111.30]	
MMP-14, ng/mL	INOCA/ANOCA	0.71 [0.29–1.04]	<0.001 * p_obstructive CAD–control_ < 0.001 *p*_control–INOCA_ = 0.02
	Obstructive CAD	0.45 [0.26–0.78]	
	Control	1.00 [0.75–1.31]	

* Statistically significant—*p* < 0.05; CAD—coronary artery disease; INOCA/ANOCA—ischemia/angina with non-obstructive coronary arteries; VEGF—vascular endothelial growth factor; TNF-α—tumor necrosis factor-α; MMP—matrix metalloproteinase.

**Table 4:** Univariate logistic regression analysis for different phenotypes of CAD.

Factor/Predictor	B	Exp (B) [95%CI]	*p*	Pseudo R-squ
Gender (male/female)	−1.409	0.244 [0.094, 0.636]	*p* = 0.004 *	0.080
Smoking (n)	0.063	1.064 [0.244, 4.641]	*p* = 0.933	0.000
Hypertension (n)	0.138	1.147 [0.210, 6.28]	*p* = 0.874	0.000
Dyslipidemia (n)	0.323	1.381 [0.138, 13.85]	*p* = 0.784	0.001
Angina pain (n)	0.642	1.899 [0.629, 5.736]	*p* = 0.255	0.012
Fasting glucose (mmol/L)	0.018	1.018 [1.004, 1.033]	*p* = 0.014 *	0.053
Myocardial infarction (n)	−1.488	0.226 [0.077, 0.662]	*p* = 0.007 *	0.073
ACE inhibitors	−1.082	0.339 [0.138, 0.831]	*p* = 0.018 *	0.049
ARB II	0.799	2.222 [0.876, 5.637]	*p* = 0.093	0.024
Beta blockers	0.386	1.471 [0.432, 5.01]	*p* = 0.536	0.003
Calcium channel blocker	0.669	1.952 [0.813, 4.691]	*p* = 0.135	0.019
Antiaggregants	−1.157	0.314 [0.066, 1.505]	*p* = 0.148	0.018
Statins	−0.776	0.460 [0.028, 7.619]	*p* = 0.588	0.002
Age (years)	0.004	1.004 [0.953, 1.058]	*p* = 0.871	0.000
BMI (kg/m^2^)	0.007	1.006 [0.903, 1.122]	*p* = 0.905	0.000
VEGF (ng/mL)	0.000	1.000 [0.998, 1.001]	*p* = 0.691	0.001
TNF-α (ng/mL)	−0.002	0.998 [0.992, 1.004]	*p* = 0.433	0.006
MMP-1 (ng/mL)	−0.046	0.955 [0.587, 1.553]	*p* = 0.853	0.000
MMP-9 (ng/mL)	−0.044	0.957 [0.906, 1.011]	*p* = 0.118	0.029
MMP-13 (ng/mL)	0.000	1.000 [1.000, 1.0]	*p* = 0.972	0.000
MMP-14 (ng/mL)	−0.025	0.975 [0.890, 1.07]	*p* = 0.600	0.003
miR-34a REU	−0.050	0.951 [0.869, 1.041]	*p* = 0.274	0.010
miR-145 REU	0.444	1.558 [1.066, 2.277]	*p* = 0.022 *	0.042
miR-222 REU	0.458	1.581 [0.422, 5.93]	*p* = 0.497	0.003

* Statistically significant—*p* < 0.05; CAD—coronary artery disease; BMI—body mass index; ACE inhibitors—angiotensin-converting enzyme inhibitors; ARB II—angiotensin II receptor blockers; VEGF—vascular endothelial growth factor; TNF-α—tumor necrosis factor-α; MMP—matrix metalloproteinase.

**Table 5:** Multivariate logistic regression analysis for CAD and INOCA/ANOCA groups.

Variables	Coef (B)	Exp (B)	*p*
miR-145 REU	0.921	2.512 [1.294, 4.875]	*p* = 0.006 *
Gender (male/female)	−1.116	0.328 [0.121, 0.889]	*p* = 0.029 *

* Statistically significant—*p* < 0.05.

**Table 2:** CAD patients’ therapy characteristics.

	INOCA/ANOCA	Obstructive CAD	*p*-Value
ACE inhibitors	18 (35.3)	47 (61.8)	0.027 *
ARB II	17(33.3)	20 (26.7)	0.123
Beta blockers	26 (86.7)	53 (81.5)	0.535
Calcium channel blockers	16 (53.3)	24 (36.9)	0.33
Antiaggregants	26 (66.7)	62 (81.5)	0.202
Anticoagulants	4 (10.2)	7 (9.2)	0.738
Antiarrhythmic drugs	3 (7.7)	8 (10.5)	1.000
HMG-CoA reductase inhibitors	29 (74.4)	63 (82.9)	0.539

INOCA/ANOCA—ischemia/angina with non-obstructive coronary arteries; ACE inhibitors—angiotensin-converting enzyme inhibitors; ARB II—angiotensin II receptor blockers; *—statistically signficant *p* < 0.05

**Table 6:** Primer sequences for RT-PCR.

miRNA	Primer Sequence
miR-34a	5′-TGGCAGTGTCTTAGCTGGTTGT-3′
miR-145	5′-TCCAGTTTTCCCAGGAATCCCT-3′
miR-222	5′-CTCAGTAGCCAGTGTAGATCCT-3′

## Data Availability

The data presented in this study are available upon request from the corresponding author. The data are not publicly available because some data sets will be used for further research.
